# A carboxylesterase-selective ratiometric fluorescent two-photon probe and its application to hepatocytes and liver tissues†
†Electronic supplementary information (ESI) available: Synthesis, additional methods, and figures (Fig. S1–S17, Tables S1 and S2). See DOI: 10.1039/c5sc05001d


**DOI:** 10.1039/c5sc05001d

**Published:** 2016-02-23

**Authors:** Sang Jun Park, Hyo Won Lee, Hye-Ri Kim, Chulhun Kang, Hwan Myung Kim

**Affiliations:** a Department of Chemistry and Department of Energy Systems Research , Ajou University , Suwon 443-749 , Korea . Email: kimhm@ajou.ac.kr ; Fax: +82-31-219-1615; b School of East–West Medical Science , Kyung Hee University , Yongin 446-701 , Korea . Email: kangch@khu.ac.kr

## Abstract

Carboxylesterases (CEs) are widely distributed enzymes in the human body that catalyze hydrolysis of various endogenous and exogenous substrates. They are directly linked to hepatic drug metabolisms and steatosis, and their regulations are important issues in pharmacological and clinical applications. In this work, we have developed an emission ratiometric two-photon probe (**SE1**) for quantitatively detecting CE *in situ*. This probe is based on a translation of intramolecular charge transfer character upon reaction with CE. It shows a sensitive blue-to-yellow emission change in response to human CE activity, easy loading into cells, insensitivity to pH and other metabolites including ROS and RNS, high photostability, and low cytotoxicity. Using live hepatocytes and liver tissues, we found that ratiometric two-photon microscopic imaging with **SE1** is an effective tool for monitoring CE activities at the subcellular level in live tissues. This probe will find useful applications in biomedical research, including studies of hepatic steatosis and drug developments.

## Abstract 2

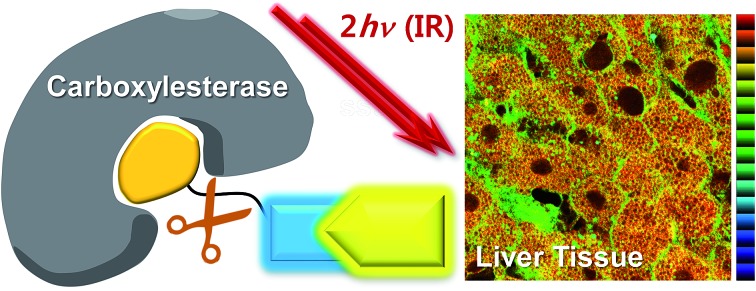
A ratiometric two-photon fluorescent probe for quantitative detection of carboxylesterase activity in live cells and tissues was reported.

## Introduction

Carboxylesterases (EC 3.1.1.1, CEs) are a multigene carboxylesterase family that catalyzes hydrolysis of a vast number of endogenous and exogenous substrates including drugs.^1^,^2^ In humans, five isozymes of CEs have been reported to date.^1^,^3^ They are widely and heterogeneously distributed in the body, with CE1 and CE2 being mainly expressed in the liver and playing major roles in drug metabolism and hepatic fat metabolism.^4^ Abnormal regulation or deficiency of CE1 and CE2 is directly linked to human diseases such as hepatic steatosis, obesity, hyperlipidemia, insulin insensitivity, and cancer.^5^–^8^ These two isoenzymes also play important roles in the modification or clearance of various drugs; substrate specificity of CE1 and 2 in the liver is an important issue in drug metabolism, as are their side-effects in the clinical setting.^9^–^11^ To understand the physiological and pharmacological roles of CE, it is crucial to develop a method for precisely monitoring CE *in situ* at the cell, tissue, and organ levels.

Small-molecule fluorescent probes such as fluorescein diacetate (FDA) have been used for measuring CE activity *in vitro* and in cells.^12^–^15^ However, most of these probes suffer from off–on response at a single detection window, pH-sensitivity, low cell loading ability, and instability in cell culture medium. Therefore, their applications for quantitative analysis of CE in live samples are limited. Recently, ratiometric probes derived from 2-(benzo[*d*]thiazol-2′-yl)phenol have been reported for detecting CE in live cells.^16^ However, their use in live cells and tissues has been impractical due to the required short excitation wavelengths (<400 nm) that may cause photodamage, artificial production of reactive oxygen species (ROS), and short tissue penetrating depth.

Two-photon microscopy (TPM) has been adopted as a powerful tool for biomedical studies because of its intrinsic localization of excitation, low photodamage, longer observation times, and greater tissue penetration depth.^17^–^19^ In combination with TPM, a ratiometric measurement based on the fluorescence intensities of a reactant and the corresponding product makes possible the quantitative imaging analysis of enzyme activity without the experimental artifacts of probe concentration or distribution, excitation laser power, detection sensitivity, and photobleaching.^20^–^31^ Hence, for quantitative measurement of the CE activity in living samples, an emission ratiometric TP probe would be advantageous.

**Scheme 1:**
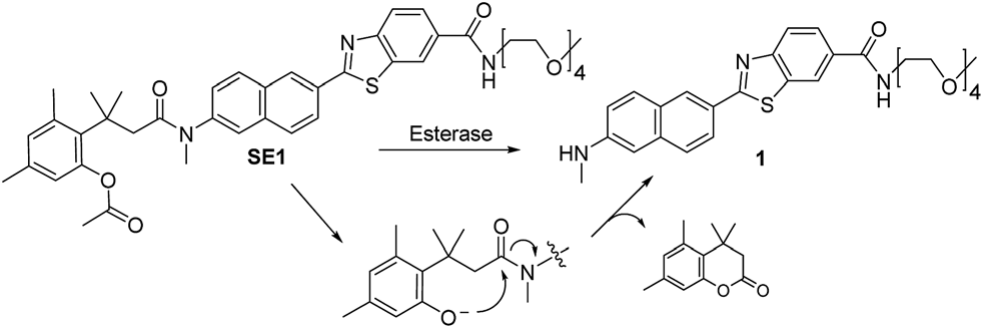
Structures of **SE1** and **1**, and the proposed mechanism of the reaction of **SE1** with esterase.

In this work, we report a ratiometric TP probe for CE (**SE1**, Scheme 1) and its application to quantitative analysis of CE activity in living hepatocytes and liver tissues. This probe is composed of a trimethyl-lock based phenyl acetate (TLPA), 3-(2-acetoxy-4,6-dimethylphenyl)-3-methylbutyric acid derivative, as the CE hydrolytic site^32^ and an electron donor–acceptor substituted TP fluorophore containing the solubilizing group 2,5,8,11-tetraoxatridecan-13-amine.^21^,^33^ TLPA is known to be stable in cell culture media compared to acetate derived FDA.^34^ The sensing process of **SE1** is based on the translation of intramolecular charge transfer (ICT) character between the probe and enzymatic reaction product. As proposed in Scheme 1, CE-mediated hydrolysis can produce the deacetylated derivative of the probe, which triggers cleavage of the amide bond through cyclization to liberate an amino group, a stronger electron donor than that for **SE1**. Eventually, the emission maximum shifts to the red region with the increased ICT character of the product (**1**). This translation can provide ratiometric analysis of enzyme activity by simultaneous monitoring of two detection windows for **SE1** and **1** using an appropriate excitation source.

## Results and discussion

The preparation of **SE1** and **1** is described in the ESI.† First of all, we investigated the spectral properties of **SE1** and **1** under physiological buffer conditions. The solubility of **SE1** and **1** in PBS buffer (10 mM, pH 7.4) was determined by a fluorescence titration method and the values were approximately 2 and 3 μM, which were sufficient to stain the cells (Fig. S1, ESI†). In an aqueous solution (10 mM PBS, pH 7.4), **SE1** and **1** displayed fluorescence maxima (*λ*_fl_) at 455 nm (*Φ* = 0.55) and 540 nm (*Φ* = 0.16) with corresponding absorption maxima (*λ*_abs_) at 332 nm (*ε* = 2.37 × 10^4^ M^–1^ cm^–1^) and 378 nm (*ε* = 2.50 × 10^4^ M^–1^ cm^–1^), respectively (Table S1, ESI†). The Stokes shift observed in **1** was larger than for **SE1** (162 *vs.* 123 nm) and may be due to the greater stabilization of the charge-transfer excited state in **1**, which contains a stronger electron-donating group.

**Fig. 1:**
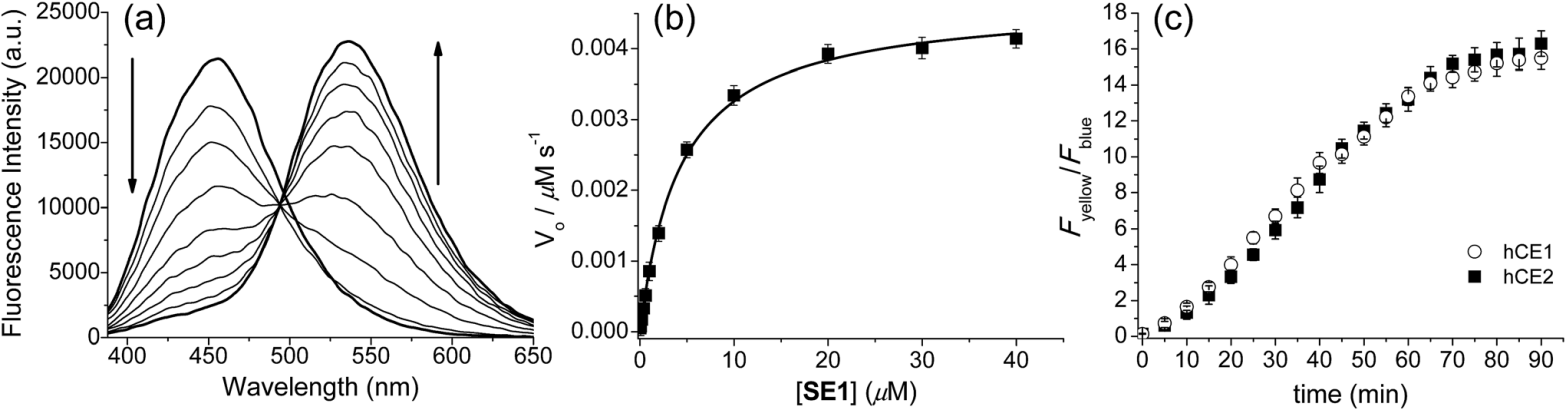
Enzymatic reaction of **SE1** with esterases. (a) Fluorescence spectra of **SE1** (1.0 μM) in PBS buffer (10 mM, pH 7.4, 37 °C) before and after the addition of 0.2 unit porcine liver esterase (PLE), measured every 1 min. (b) Plot of *V*_0_*versus* various concentrations of **SE1**. (c) Time course of the ratio (*F*_yellow_/*F*_blue_) of **SE1** (1.0 μM) after addition of human CE1 and CE2 (approximately 10 units) in PBS buffer.

Upon treatment with porcine liver esterase (PLE), whose activity is known to be similar to human CE1,^35^ the emission spectra of **SE1** in PBS buffer (10 mM, pH 7.4, 37 °C) showed a gradual decrease at 455 nm with a concomitant increase at 540 nm, the *λ*_fl_ for **1** (Fig. 1a). The HPLC analysis confirmed that **1** is the only major product (Fig. S2, ESI†) in the enzymatic reaction of **SE1**. The ratio of the integrated emission intensities (*F*_yellow_/*F*_blue_) between 420–470 nm (*F*_blue_) and 520–570 nm (*F*_yellow_) increased 118-fold upon PLE reaction. Further, the plot of the *F*_yellow_/*F*_blue_*versus* the PLE concentration ranging from 0 to 25 nM showed a linear relationship (Fig. S3, ESI†), indicating that **SE1** can detect PLE at concentrations as low as 0.5 nM. According to the Michaelis–Menten kinetics, the apparent specificity constant of **SE1** for the PLE-catalyzed reaction was determined to be *k*_cat_/*K*_m_ = 2.1 × 10^5^ M^–1^ s^–1^, where *K*_m_ = 4.33 ± 0.22 μM (Fig. 1b and Table S2, ESI†). This value is more than 100-fold higher than that of the diacetylated trimethyl lock containing rhodamine derivative.^34^ In addition, the activities of **SE1** by human CE1 (hCE1) and CE2 (hCE2) under the same conditions were almost equal as shown in Fig. 1c. Therefore, **SE1** is a sensitive fluorescent probe for ratiometric detection of CE activity in physiological buffer.

**Fig. 2:**
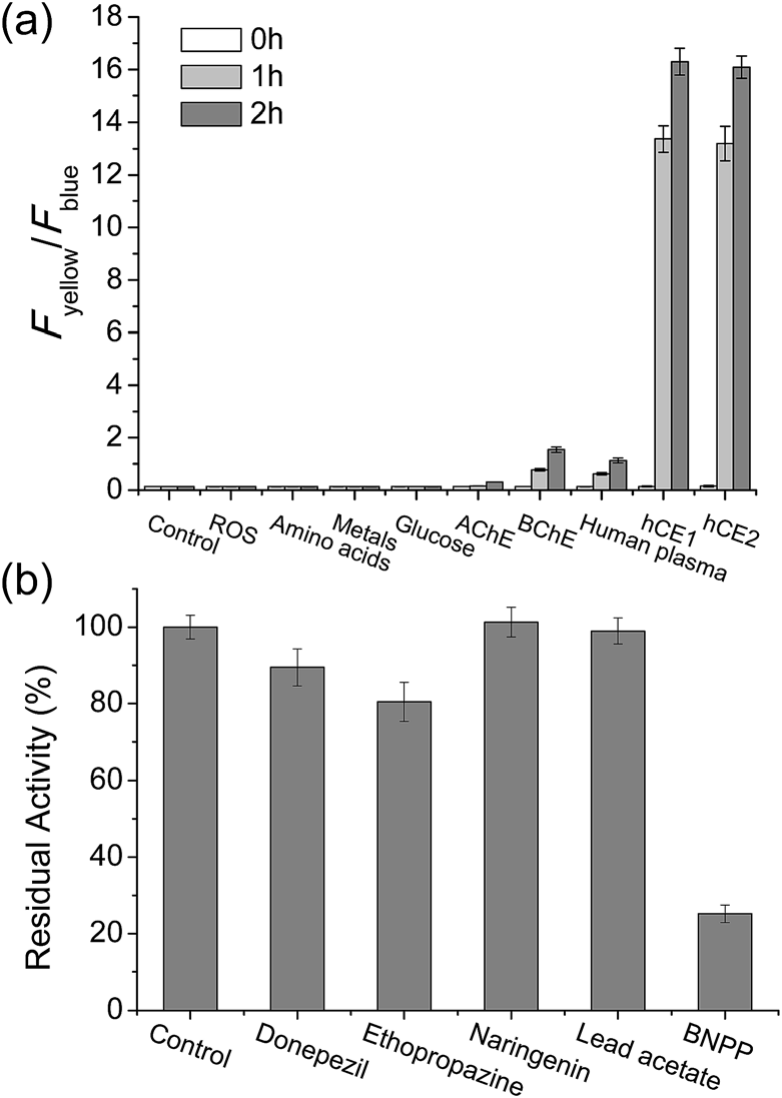
**SE1** selectivity experiments. (a) Fluorescence responses of 1 μM **SE1** to ROS (H_2_O_2_, TBHP, OCl^–^, O_2_^–^, NO, ˙O^*t*^Bu, ˙OH, ONOO^–^), amino acids (Cys, Glu, Gly, Val, Thr, Tyr, Trp, Ser, Phe, Met, Leu, Ile, Asp, Lys, His), metals (Na^+^, K^+^, Ca^2+^, Mg^2+^), glucose, AChE, BChE, human plasma, hCE1, and hCE2. Bars represent the integrated fluorescence ratios (*F*_yellow_/*F*_blue_) at 0 to 2 hours after the addition of each species. (b) Fluorescence response of **SE1** to various inhibitors in homogenized HepG2 cells (donepezil for AChE, ethopropazine for BChE, naringenin for PON1, lead acetate for PON2, and BNPP for CE). Bars represent the relative fluorescence intensity after 15 min incubation with each inhibitor.

To validate that **SE1** is a reliable CE substrate, its selectivity was measured under various conditions. The ratio of **SE1** over the pH range from 3.5 to 10.0 was almost the same (Fig. S4, ESI†) with no interference from other metabolites including various ROS, amino acids, sugar, and metal ions (Fig. 2a). **SE1** also showed good selectivity for CE over acetylcholinesterase (AChE), butyrylcholinesterase (BChE), and human plasma, which contained various proteins including paraoxonase (PON).^36^ To confirm these results, we further conducted inhibition assays in homogenized HepG2 cells by using selective esterase inhibitors. Upon incubation with bis(4-nitrophenyl) phosphate (BNPP), a well-known inhibitor for CE,^37^ the enzymatic reaction of **SE1** was mostly inhibited in comparison to the control, while other inhibitors for AChE, BChE, PON1, and PON2 showed minimum inhibition of this reaction (Fig. 2b). Then, the non-carboxylesterase dependent hydrolysis of **SE1** was measured in the Minimum Essential Medium (MEM) with 10% Fetal Bovine Serum (FBS) and then was compared with that of FDA, a common fluorescent substrate for esterase. In contrast to FDA, which is quickly hydrolyzed (more than 70% within 1 h), less than 20% of **SE1** was hydrolyzed even after 2 h, implying that **SE1** would show much less background reactivity than FDA would under physiological conditions (Fig. S5, ESI†). Therefore, the results suggested that **SE1** is a ratiometric fluorescent substrate for CE with high sensitivity and low background reactivity, and minimum interference from pH and metabolites in the biological systems.

**Fig. 3:**
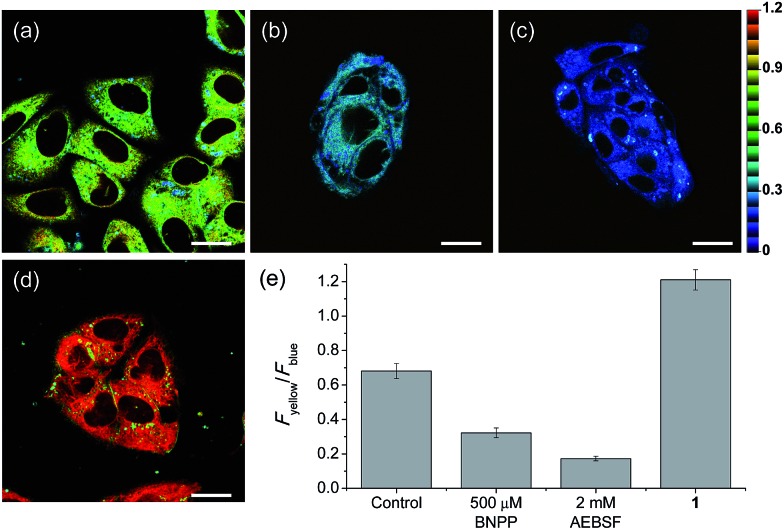
Pseudocolored ratiometric TPM images of HepG2 cells incubated with (a) **SE1** (2 μM) and (d) **1** (2 μM). HepG2 cells were pretreated with (b) bis(4-nitrophenyl) phosphate (BNPP, 500 μM) and (c) 4-(2-aminoethyl)benzenesulfonyl fluoride hydrochloride (AEBSF, 2 mM) for 30 min before labeling with **SE1**. (e) Average *F*_yellow_/*F*_blue_ intensity ratios in the TPM images. Images were acquired using 740 nm excitation and emission windows of 420–470 nm (blue) and 520–570 nm (yellow). Scale bars = (a and b) 16, and (c and d) 19 μm.

We then performed a TPM imaging study using HepG2 cells, a human hepatocyte cell line, labeled with **SE1** without any other complicated loading techniques. The TPM images of **SE1**-labeled HepG2 cells show strong TP excited fluorescence (TPEF) signals (Fig. 3), which may be attributed to the efficient cellular uptake and significant TP action cross section (*Φδ*_max_) value of the probe (Fig. S6 and Table S1, ESI†). Additionally, **SE1** showed high photostability in the cells and negligible cytotoxicity under the imaging conditions as evidenced by cell viability tests using the MTS and CCK-8 assays (Fig. S7 and S8, ESI†).

Upon excitation at 740 nm, the average emission ratios (*F*_yellow_/*F*_blue_) of HepG2 cells incubated with **SE1** and **1** for 30 min were 0.68 and 1.21, respectively (Fig. 3). The ratio of **1** was used as a standard for completion of the ester hydrolysis. The ratios for HeLa, A431, and Raw 264.7 cells incubated with **SE1** for 30 min were 0.24, 0.54, and 0.56, respectively (Fig. S9, ESI†). These values were significantly lower than for HepG2 cells, while the ratios of **1** in these cells were nearly identical to 1.21, pointing to the higher CE activity in HepG2 cells.

**Fig. 4:**
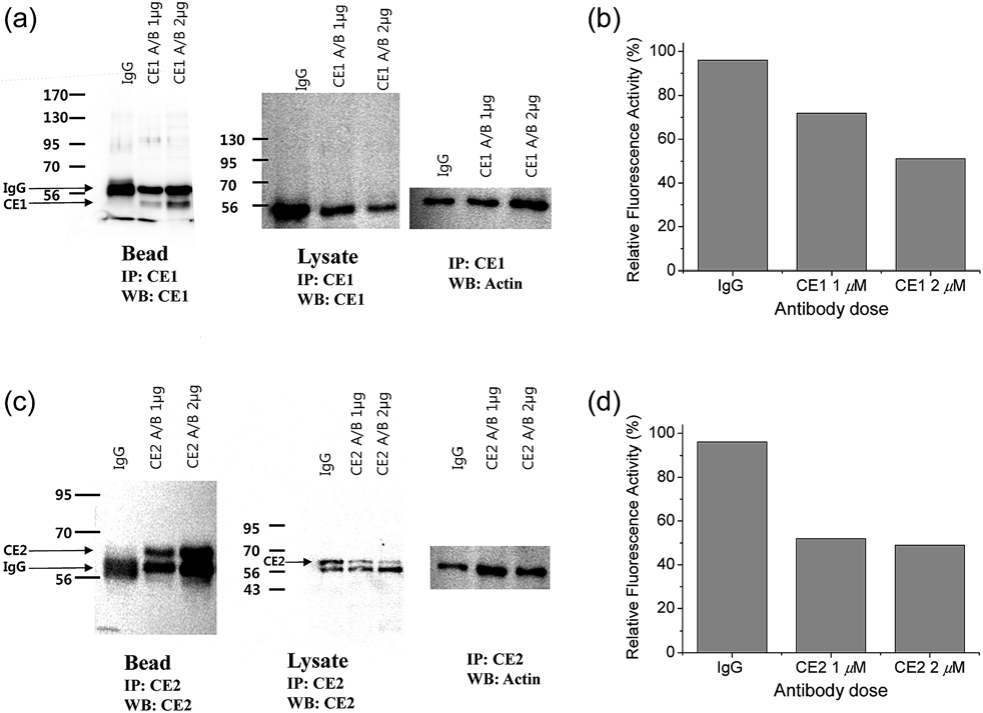
(a and c) Western blot analysis of the homogenates from HepG2 cells incubated with (a) CE1 antibody and (c) CE2 antibody using IgG antibody conjugated protein-A/G agarose bead. (b and d) Response of 1 μM **SE1** in the homogenates after immunoprecipitation with (b) CE1 antibody and (d) CE2 antibody.

To pursue whether CE caused the high emission ratios (*F*_yellow_/*F*_blue_) in HepG2 cells, we performed a control experiment with well-known inhibitors of CE, BNPP and 4-(2-aminoethyl) benzenesulfonyl fluoride hydrochloride (AEBSF).^37^,^38^ As shown in Fig. 3, the emission ratios (*F*_yellow_/*F*_blue_) in HepG2 cells with **SE1** decreased by approximately 50% and 25% upon BNPP and AEBSF treatments, respectively. These data indicate that the high ratio may be attributed to CE activities in the cells. To confirm this observation, immunoprecipitation-mediated CE depletions were used. CE1 and CE2 are the two major CE isozymes in liver tissues and can be selectively depleted from the HepG2 homogenates through immunoprecipitation using the corresponding antibody, and their depletions may be confirmed by western blot analysis (Fig. 4a and c). The ratio (*F*_yellow_/*F*_blue_) of **SE1** in the HepG2 homogenates with CE-depletion was decreased by 45% for CE1 and 47% for CE2, as compared to that of the undepleted homogenate (Fig. 4b and d). These results revealed that the high emission ratios (*F*_yellow_/*F*_blue_) in Fig. 3 are contributed by CE1 and CE2 activities in the cells.

**Fig. 5:**
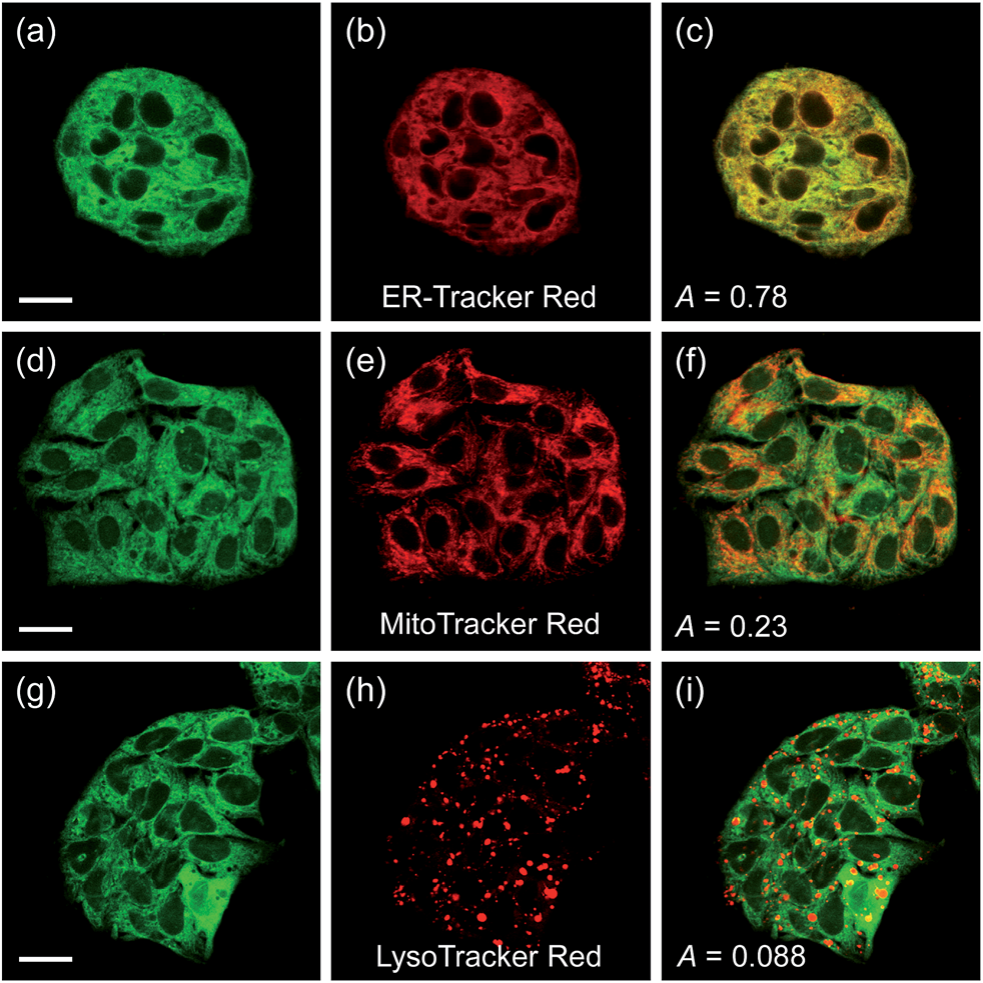
(a, d, and g) TPM and (b, e, and h) OPM images of HepG2 cells co-labeled with **SE1** (2 μM) and organelle trackers. (c, f, and i) Merged images. Excitation wavelengths for TPM and OPM are 740 nm and 552 nm, respectively. Scale bars = (a and d) 19 and (g) 23 μm.

Another issue is the subcellular affiliation of the high ratio value in HepG2 cells because CE1 and CE2 are known to be highly expressed and localized in endoplasmic reticulum (ER).^39^ To access this issue, the ratiometric images were compared with those of the well-known organelle trackers for ER, mitochondria, and lysosomes (Fig. 5). The results revealed that the image with **SE1** merged quite well with the image of the ER tracker, but poorly with those for the mitochondria and lysosome trackers, where the Pearson's colocalization coefficients (*A*) of **SE1** were 0.78, 0.23, and 0.09 for the ER, mitochondria, and lysosome trackers, respectively. This outcome agreed well with the CE activity in HepG2 cells being mainly located in the ER and further supported the conclusion that **SE1** is a selective substrate for CE in the cells. Similar results were observed with HeLa, A431, and RAW 264.7 cells (Fig. S10–12, ESI†).

**Fig. 6:**
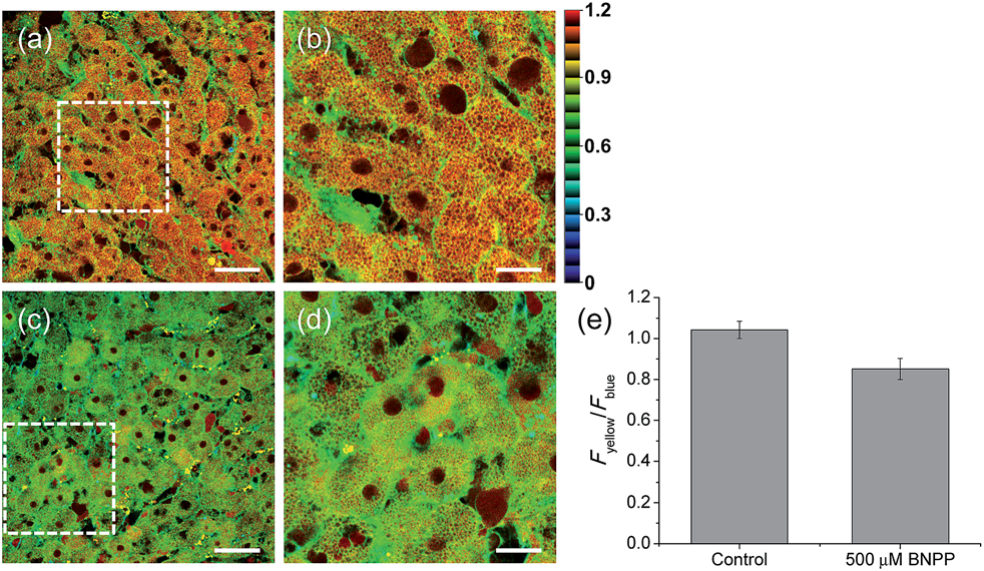
Pseudocolored ratiometric TPM images (*F*_yellow_/*F*_blue_) of rat liver tissues stained with 20 μM **SE1** for 2 h (a) before and (c) after pretreatment with 500 μM BNPP for 1 h before labeling with **SE1**. (b and d) Higher magnification images of (a) and (c). Images were acquired at depths of approximately 300 μm using a 740 nm excitation source and emission windows of 420–470 nm (*F*_blue_) and 520–570 nm (*F*_yellow_). Scale bars = (a and c) 48 and (b and d) 19 μm. (e) The average emission ratio (*F*_yellow_/*F*_blue_) in (a and c).

Finally, we used our probe to visualize the distribution and activity of CE in liver tissues. The liver was taken from a 2 month-old male Sprague Dawley rat and incubated with **SE1** for 2 h. The TPM images of **SE1**-labeled rat liver tissues clearly showed the distribution of CE activity through individual cells at a depth of about 300 μm (Fig. 6a and b). The average emission ratio (*F*_yellow_/*F*_blue_) in the liver tissue was found to be 1.04, which is a significantly higher level than that for HepG2 cells (Fig. 6e). This result might reflect the different activation level between humans and rats, as a similar result has been reported.^40^,^41^ After BNPP treatment for 1 h, the value decreased to 0.85 (Fig. 6c–e). These data indicate that **SE1** is capable of selectively monitoring CE activity deep inside of tissues using ratiometric TPM imaging.

## Conclusions

In this work, we have developed **SE1**, a new emission ratiometric TP probe that can quantitatively detect hCE activity in live cells and tissues. This probe shows a significant TP cross section, a marked blue-to-yellow emission color change in response to hCE, easy loading into cells, insensitivity to pH in the physiological range and to other metabolites including ROS and RNS, high photostability and low cytotoxicity. In addition, we found that ratiometric TPM imaging using **SE1** is an effective tool for precisely monitoring hCE activities at the subcellular level in live tissues. This probe will find useful applications in biomedical research, including studies of hepatic steatosis and drug developments.

## Experimental section

### Spectroscopic measurements

Absorption spectra were recorded on an S-3100 UV-Vis spectrophotometer and fluorescence spectra were obtained with a FluoroMate FS-2 fluorescence spectrophotometer with a 1 cm standard quartz cell. The fluorescence quantum yield was determined by using 9,10-diphenylanthrancene (*Φ* = 0.93 in cyclohexane) as the reference by the literature method.^42^

### Enzymatic kinetics assays

Enzymatic kinetics experiments were performed by using a FluoroMate FS-2 fluorescence spectrophotometer with a 1 cm standard quartz cell. Various concentrations of **SE1** (0–40 μM) were prepared in PBS buffer solution (10 mM, pH = 7.4). PLE enzyme was added to a final concentration of 0.88 μg mL^–1^, and the fluorescence intensity was collected at 455 nm (*λ*_ex_ = 373 nm) with 1 min intervals from 0 to 30 min at 37 °C. The kinetic parameters of the Michaelis–Menten equation were calculated using a hyperbolic function by the nonlinear fitting algorithm (OriginPro 8.0).

### Measurement of two-photon cross section

The two-photon cross section (*δ*) was determined by using the femtosecond (fs) fluorescence measurement technique as described.^43^**SE1** (1.0 × 10^–6^ M) was dissolved in PBS buffer (10 mM, pH = 7.4) and the two-photon induced fluorescence intensity was measured at 720–880 nm by using rhodamine 6G, whose two-photon property has been well characterized in the literature, as the reference.^44^ The intensities of the two-photon induced fluorescence spectra of the reference and sample emitted at the same excitation wavelength were determined. The TPA cross section was calculated by using following equation:
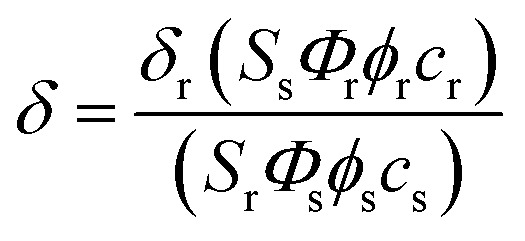
where the subscripts s and r stand for the sample and reference molecules. The intensity of the signal collected by a CCD detector was denoted as *S*. *Φ* is the fluorescence quantum yield. *φ* is the overall fluorescence collection efficiency of the experimental apparatus. The number density of the molecules in solution was denoted as *c*. *δ*_r_ is the TPA cross section of the reference molecule. Fig. S6, ESI†, shows the two-photon spectra of **SE1** and **1** in PBS buffer (10 mM, pH = 7.4).

### Selectivity assay

Each species (200 μM ROS, 1 mM amino acids and glucose, 10 μg L^–1^ AChE, 20 U L^–1^ BChE, 0.1% human plasma, 5 μg mL^–1^ hCE1 and hCE2)^16^ was administered to 1 μM **SE1** in PBS buffer (10 mM, pH 7.4) and the fluorescence spectra were acquired as time. Sample preparations are as below.

ROS: H_2_O_2_, *tert*-butylhydroperoxide (TBHP), and sodium hypochlorite (NaOCl) were from 30%, 70%, 5% aqueous solutions, respectively. Hydroxyl radicals (˙OH), and *tert*-butoxy radicals (˙O^*t*^Bu) were generated by reaction of 1 mM Fe^2+^ with 200 μM H_2_O_2_ or TBHP, respectively. Nitric oxide (NO) was used from a stock solution (1.9 mM), prepared by purging PBS buffer (10 mM, pH 7.4) with N_2_ gas for 30 min, followed by NO (99.5%) for 30 min. Superoxide (O_2_^–^) was delivered from KO_2_. Peroxynitrite from stock solution was used (10 mM in 0.3 M NaOH).

Amino acids: l-amino acids (Ala, Cys, Glu, Gly, Val, Thr, Tyr, Trp, Ser, Phe, Met, Leu, Ile, Asp, Lys, His) were purchased from Sigma-Aldrich (LAA21).

Glucose was purchased from Sigma-Aldrich (G7528). Three independent experiments for each species were performed.

AChE: acetylcholinesterase was purchased from Sigma-Aldrich (C2888).

BChE: butyrylcholinesterase was purchased from Sigma-Aldrich (C7512).

Human plasma was purchased from Sigma-Aldrich (H4522).

hCEs: carboxylesterase 1 human was purchased from Sigma-Aldrich (E0287) and carboxylesterase 2 human was purchased from Sigma-Aldrich (E0412).

### Inhibitor assay

HepG2 cells were washed with PBS and homogenized using a bullet blender (Next advance) in 50 mM Tris–HCl (pH 7.4), 150 mM NaCl, 0.5% Triton X-100, 1 mM EDTA, and protease inhibitor cocktail (all reagents are from Sigma) for 30 min in ice. Centrifugation of the homogenate at 10 000 rpm for 15 min gave a clear supernatant which was collected into a new tube. 100 μg protein of the supernatant was incubated with 10 μM of each inhibitor (donepezil hydrochloride monohydrate for the AChE inhibitor,^45^ ethopropazine hydrochloride for the BChE inhibitor,^46^ naringenin for the PON1 inhibitor,^47^ lead acetate for the PON2 inhibitor,^48^ and bis(4-nitrophenyl) phosphate (BNPP) for the CE inhibitor).^37^ The relative fluorescence intensity of 3 μM **SE1** with each inhibitor was acquired after 15 min incubation. The excitation wavelength was 405 nm.

### Cell culture

All the cells were passed and plated on glass-bottomed dishes (NEST) for two days before imaging. They were maintained in a humidified atmosphere of 5/95 (v/v) of CO_2_/air at 37 °C. The cells were treated and incubated with 2 μM **SE1** and **1** at 37 °C under 5% CO_2_ for 30 min, washed three times with phosphate buffered saline (PBS; Gibco), and then imaged after further incubation in colorless serum-free media for 30 min. The culture mediums for each cell are as below.

HepG2 cells (ATCC, Manassas, VA, USA): MEM (WelGene Inc, Seoul, Korea) supplemented with 10% FBS (WelGene), penicillin (100 units per mL), and streptomycin (100 μg mL^–1^).

HeLa human cervical carcinoma cells (ATCC, Manassas, VA, USA): MEM (WelGene Inc, Seoul, Korea) supplemented with 10% FBS (WelGene), penicillin (100 units per mL), and streptomycin (100 μg mL^–1^).

A431 cells (ATCC, Manassas, VA, USA): RPMI1640 (WelGene Inc, Seoul, Korea) supplemented with 10% FBS (WelGene), penicillin (100 units per mL), and streptomycin (100 μg mL^–1^).

Raw 264.7 cells (ATCC, Manassas, VA, USA): DMEM (WelGene Inc, Seoul, Korea) supplemented with 10% FBS (WelGene), penicillin (100 units per mL), and streptomycin (100 μg mL^–1^).

### Immunoprecipitation and western blot analysis

For immunoprecipitation, the HepG2 cells were washed with PBS and homogenized using bullet blender (Next advance) in 50 mM Tris–HCl (pH 7.4), 150 mM NaCl, 0.5% Triton X-100, 1 mM EDTA, and protease inhibitor cocktail (all reagents are from Sigma) for 30 min in ice. Centrifugation of the homogenate at 10 000 rpm for 15 min gave a clear supernatant which was collected into a new tube. 500 μg protein of the supernatant was incubated with anti-CES1 (sc-365248) or anti-CES2 (sc-100685), or a control IgG (sc-2025) antibody and protein A/G agarose bead (GenDEPOT) for overnight at 4 °C. The antibody-conjugated agarose beads were centrifuged at 2000 rpm for 2 min and washed three times with homogenization buffer. The proteins from the beads and the supernatant were used for western blot analysis. For western blot analysis, the proteins from SDS-PAGE were transferred to a PVDF membrane (Bio-Rad). The membrane was treated with in 5% non-fat milk for 2 h and was incubated with anti-CES1, anti-CES2, or anti-actin antibodies overnight at 4 °C. A goat anti-mouse IgG-HRP (sc-2005) was used as the secondary antibody, and the bands were visualized by Ez-Western Lumi Plus (ATTO). The antibodies in the western blot analysis were purchased from Santa Cruz.

### Two-photon fluorescence microscopy

Two-photon fluorescence microscopy images of **SE1**-labeled cells and tissues were obtained with spectral confocal and multiphoton microscopes (Leica TCS SP8 MP) with ×10 dry, ×40 oil and ×100 oil objectives, numerical aperture (NA) = 0.30, 1.30, and 1.30. The two-photon fluorescence microscopy images were obtained with a DMI6000B Microscope (Leica) by exciting the probes with a mode-locked titanium-sapphire laser source (Mai Tai HP; Spectra Physics, 80 MHz, 100 fs) set at wavelength 740 nm and output power 2901 mW, which corresponded to approximately 1.31 × 10^8^ mW cm^–2^ average power in the focal plane. Live cell imaging was performed using live cell incubator systems (Chamlide IC; Live Cell Instrument) for a stable cell environment by maintaining proper temperature, humidity and pH over the long term. To obtain images in the 420–470 nm (*F*_blue_) and 520–570 nm (*F*_yellow_) ranges, internal PMTs were used to collect the signals in an 8 bit unsigned 512 × 512 and 1024 × 1024 pixels at 400 and 200 Hz scan speed, respectively. Ratiometric image processing and analysis was carried out using MetaMorph software.

### Co-localization experiments

Co-localization experiments were conducted by co-staining the HepG2, HeLa, A431, and Raw 264.7 cells with appropriate combinations of **SE1** (2.0 μM) and 1.0 μM of each commercial organelle tracker (LysoTracker Red DND-99 for lysosome, MitoTracker Red FM for mitochondria, ER-Tracker Red for endoplasmic reticulum) for 30 min. TPM and OPM images were obtained by collecting the emissions at 520–570 nm (**SE1**, *λ*_ex_ = 740 nm) and 600–650 nm (organelle trackers, *λ*_ex_ = 552 nm), respectively. The background images were corrected, and the distribution of pixels in the TPM and OPM images acquired in the green and red channels, respectively, was compared by using a scattergram. The Pearson's colocalization coefficient (*A*) was calculated by using the AutoQuant X2 program.

### Preparation and staining of fresh rat liver slices

Rat liver slices were prepared from the liver of an 8 week old Sprague Dawley rat. The liver slices were cut into 800 μm thicknesses using a vibrating-blade microtome in PBS buffer. Slices were incubated with 20 μM **SE1** in PBS bubbled with 95% O_2_ and 5% CO_2_ for 2 h at 37 °C. Slices were then washed three times with PBS and transferred to glass-bottomed dishes (NEST) and observed in a spectral confocal multiphoton microscope. The TPM images were obtained at about 300 μm depth.

## Supplementary Material

Supplementary information
