# Supplementary material for: A carboxylesterase-selective ratiometric fluorescent two-photon probe and its application to hepatocytes and liver tissues
Source: Chem Sci. 2016 Feb 23;7(6):3703–9. doi: 10.1039/c5sc05001d (PMC6008934; doi:10.1039/c5sc05001d)
Supplement: Supplementary file 1 [file SC-007-C5SC05001D-s001.pdf]

Electronic Supplementary Material (ESI) for Chemical Science.
This journal is © The Royal Society of Chemistry 2016

Supporting Information for

"A Carboxylesterase-Selective Ratiometric Fluorescent Two-Photon Probe and Its Application to Hepatocytes and Liver Tissues"

Sang Jun Park,[1] Hyo Won Lee,[1] Hye-Ri Kim,[2] Chulhun Kang,*[2] and Hwan Myung Kim*[1]

[1]*Department of Energy Systems Research, Ajou University, Suwon 443-749, Korea.*

Fax: +82-31-219-1615, Email: kimhm@ajou.ac.kr

[2]*School of East-West Medical Science, Kyung Hee University, Yongin 446-701, Korea. 443-721, Korea.*

Email: kangch@khu.ac.kr

Table of Contents

Page

Synthesis of SE1 ...... S3

Water solubility S4

Stability in cell medium S7

Photostability S8

Cell viability S9

Figure S1. (a) One-photon fluorescence spectra and (b) plot of fluorescence intensity against the concentration of **SE1** in PBS buffer (10 mM, pH 7.4). The excitation wavelength was 332 nm S4

Figure S2. HPLC traces of (a) **SE1**, (b) the reaction product between **SE1** and PLE, (c) **1**. HPLC conditions: 1.0 mL/min flow rate, 5% B to 100% B over 30 min, detected at 370 nm. Solvent A is water and solvent B is acetonitrile. Peaks at 16.4 min and 13.6 min correspond to **SE1** and **1**, respectively S5

Figure S3. Plot of the F_{yellow}/F_{blue} ratios for **SE1** vs [PLE] in PBS buffer (10 mM, pH 7.4). Each data was acquired 10 min after PLE addition at 37℃. The detection limit was calculated with 3σ/k; where σ is the standard deviation of blank measurement, k is the slope in Fig. S3 ...... S6

Figure S4. Effect of pH on the fluorescence intensity ratios (F_{yellow}/F_{blue}) for **SE1** in universal buffer (0.1 M citric acid, 0.1 M KH_2PO_4, 0.1 M $Na_2B_4O_7$, 0.1 M Tris, 0.1 M KCl) at 25 °C. The excitation wavelength was 373 nm (n = 3) S6

Figure S5. Comparison of the time course of the normalized fluorescence intensity between 1.0 µM **SE1** (red) and 1.0 µM FDA (black) in MEM medium containing 10% (v/v) FBS S7

Figure S6. Two-photon action spectra of **SE1** and **1** in PBS buffer (10 mM, pH 7.4). The estimated uncertainties for the two-photon action cross section values ($\delta\Phi$) are ± 15 %. Data for **1** was taken from Ref. 3 ...... S7

Figure S7. (a) TPM images of HepG2 cells labeled with **SE1**. (b) The relative TPEF intensity from A–C in Figure (a) as a function of time. The digitized intensity was recorded with 2.00 sec intervals for the duration of one hour using *xyt* mode. The TPEF intensities

were collected at 420–570 nm upon excitation at 740 nm with femto-second pulses. Scale bar = 48 μm. Cells shown are representative images from replicate experiments (n = 3).......... S8
Figure S8. Viability of HepG2 cells in the presence of **SE1** as measured by using MTS and CCK-8 assays. The cells were incubated with 0–20 μM **SE1** for 2 h. Six independent experiments are performed.. S9
Figure S9. Pseudocolored ratiometric TPM images (F_{yellow}/F_{blue}) of (a) HeLa, (b) A431, (c) Raw 264.7 and (d) HepG2 cells incubated with **SE1** (2.0 μM) for 30 min. (e) Average F_{yellow}/F_{blue} intensity ratios in (a-d). Images were acquired using 740 nm excitation and emission windows of 420–470 nm (blue) and 520–570 nm (yellow). Scale bars = (a) 27 and (b-d) 19 μm. Cells shown are representative images from replicate experiments (n = 10)........ S10
Figure S10. (a,d,g) TPM, (b,e,h) OPM images of HeLa cells co-labeled with (a,d,g) **SE1** (2 μM) and (b,e,h) organelle trackers. (c,f,i) Merged images. Excitation wavelengths for TPM and OPM are 740 nm and 552 nm, respectively. Scale bars = (a) 24 and (d, g) 20 μm.. S11
Figure S11. (a,d,g) TPM, (b,e,h) OPM images of A431 cells co-labeled with (a,d,g) **SE1** (2 μM) and (b,e,h) organelle trackers. (c,f,i) Merged images. Excitation wavelengths for TPM and OPM are 740 nm and 552 nm, respectively. Scale bars = (a, g) 21 and (d) 19 μm............. S11
Figure S12. (a,d,g) TPM, (b,e,h) OPM images of Raw 264.7 cells co-labeled with (a,d,g) **SE1** (2 μM) and (b,e,h) organelle trackers. (c,f,i) Merged images. Excitation wavelengths for TPM and OPM are 740 nm and 552 nm, respectively. Scale bars = (a, g) 15 and (d) 24 μm.... S12
Figure S13. ^{1}H-NMR spectrum (400 MHz) of **SE1** in $CDCl_3$.. S13
Figure S14. ^{13}C-NMR spectrum (100 MHz) of **SE1** in $CDCl_3$... S13
Figure S15. HRMS spectrum of **SE1**... S14
Figure S16. ^{1}H-NMR spectrum (400 MHz) of **1** in $CDCl_3$.. S15
Figure S17. HRMS spectrum of **1**.. S15
Table S1. Photophysical data for **SE1** and **1** in buffer.. S5
Table S2. Kinetic parameters for **SE1** with PLE... S6

Synthesis of SE1. Compound **A**, **B**, and **1** were prepared by the literature methods[1-3] and synthesis of **SE1** and detail for **1** are described below.

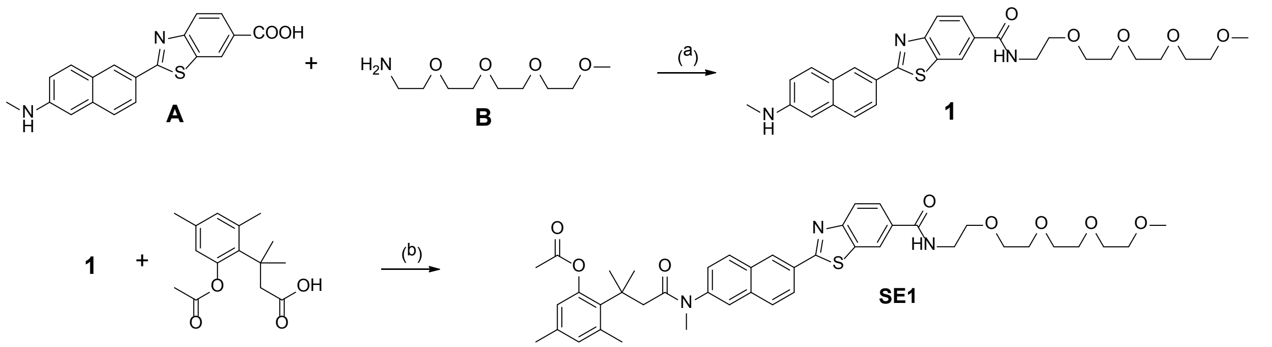

Scheme S1. Synthesis of **SE1**. *Reagents and conditions:* (a) DCC, HOBt, DMF, N_2, rt, 10h. (b) EDC, pyridine, DMF, N_2, rt, 48h.

Synthesis of **1.** To a stirred solution of **A** (250 mg, 0.75 mmol) in dry DMF (10 mL), *N*,*N'*-dicyclohexylcarbodiimide (DCC, 232 mg, 1.12 mmol) and 1-hydroxybenzotriazole (HOBt, 152 mg, 1.12 mmol) were added and the reaction mixture was stirred for one hour at room temperature under nitrogen atmosphere. Then, 2,5,8,11-tetraoxatridecan-13-amine (**B**, 156 mg, 0.75 mmol) was added to the reaction mixture and further reaction was continued for 12 h. After completion of the reaction, solvent was evaporated and the crude mixture was dissolved in CH_3CN. Precipitate dicyclohexylurea was removed by filtration and the filtrate was then concentrated under reduced pressure. The product was purified by column chromatography using 5 % CH_3OH in EtOAc as eluent to obtain **1** as a yellow semi-solid (280 mg, 72 %); ^1H NMR ($CDCl_3$, 400 MHz): δ (ppm) 8.40 (s, 1H), 8.34 (s, 1H), 8.01 (d, *J* = 8.8 Hz, 1H), 8.00 (d, *J* = 8.8 Hz, 1H), 7.90 (dd, *J* = 8.4, 2.0 Hz, 1H), 7.66 (d, *J* = 8.8 Hz, 1H), 7.64 (d, *J* = 8.4 Hz, 1H), 7.35 (brs, NH-amide, 1H), 6.87 (dd, *J* = 8.8, 2.0 Hz, 1H), 6.72 (d, *J* = 2.0 Hz, 1H), 4.29 (brs, 1H), 3.70-3.61 (m, 12H), 3.58 (t, *J* = 4.8 Hz, 2H), 3.49 (t, *J* = 4.8 Hz, 2H), 3.31 (s, 3H), 2.92 (s, 3H); ^{13}C NMR ($CDCl_3$, 100 MHz): δ (ppm) 171.4, 167.2, 156.2, 148.7, 137.3, 135.1, 131.1, 130.1, 128.0, 126.8, 126.7, 126.6, 125.3, 125.0, 122.4, 121.5, 118.9, 103.4, 72.1, 70.8, 70.7, 70.5, 70.2, 59.2, 40.4, 30.8; HRMS (FAB^+): *m/z* calculated for $[C_{28}H_{33}O_5N_3S]^+$: 524.2220, found: 524.2219.

Synthesis of **SE1**: To a stirred solution of 3-(2-acetoxy-4,6-dimethylphenyl)-3-methylbutyric acid (48 mg, 0.182 mmol, Aldrich, #756377) in 10 mL of a 1:1 mixture of dry pyridine/DMF, *N*-(3-dimethylaminopropyl)-*N'*-ethylcarbodiimide hydrochloride (EDC, 35 mg, 0.182 mmol) was added and the reaction mixture was stirred for one hour at room temperature under nitrogen atmosphere. Then, **1** (48 mg, 0.092 mmol) was added to the reaction mixture and further reaction was continued for 48 h. After completion of the reaction, solvent was evaporated, the reaction mixture was washed with brine (30 mL) and DCM (30 mL × 3) and finally dried over anhydrous sodium sulfate. The

product was purified by column chromatography using 10 % CH_3OH in $CHCl_3$ as eluent to obtain **SE1** as a pale yellow semi solid (21 mg, 30 %); ^{1}H NMR ($CDCl_3$, 400 MHz): δ (ppm) 8.59 (s, 1H), 8.53 (d, *J* = 1.6 Hz, 1H), 8.26 (dd, *J* = 8.8 Hz, 1.6Hz, 1H), 8.12 (d, *J* = 8.8 Hz, 1H), 7.98 (dd, *J* = 8.4, 1.6 Hz, 1H), 7.97 (d, *J* = 8.8 Hz, 1H), 7.86 (d, *J* = 8.4 Hz, 1H), 7.42 (s, 1H), 7.38 (brs, NH-amide, 1H), 7.25 (dd, *J* = 8.8, 1.6 Hz, 1H), 6.68 (s, 1H), 6.53 (s, 1H), 3.73-3.64 (m, 12H), 3.62-3.60 (m, 2H), 3.52-3.50 (m, 2H), 3.32 (s, 3H), 3.24 (s, 3H), 2.75 (s, 2H), 2.28 (s, 3H), 2.23 (s, 3H), 2.01 (s, 3H), 1.51 (s, 6H); ^{13}C NMR ($CDCl_3$, 100 MHz): δ (ppm) 171.1, 170.1, 169.8, 166.9, 156.1, 149.6, 143.6, 138.5, 135.9, 135.4, 135.2, 134.1, 132.4, 132.0, 131.5, 130.6, 129.0, 127.6, 127.1, 126.0, 125.5, 123.1, 121.8, 72.2, 70.8, 70.5, 70.1, 59.3, 46.1, 40.4, 40.1, 37.9, 32.4, 30.1, 25.7, 25.6, 22.2, 22.1, 20.7, 20.6; HRMS (FAB$^+$): *m/z* calculated for $[C_{43}H_{51}O_8N_3SNa]^+$: 792.3289, found: 792.3289.

Water solubility. Small amount of dye was dissolved in DMSO to prepare the stock solutions (1.0×10^{-2} M). The solution was diluted to 1.0×10^{-5} ~ 5.0×10^{-8} M and added to a cuvette containing 3.0 mL of PBS buffer (10 mM, pH 7.4) by using a micro syringe. In all cases, the concentration of DMSO in buffer was maintained to be 0.1 %.[4] The plot of fluorescence intensity against the dye concentration were linear at low concentration and showed downward curvature at higher concentration (Figure S1). The maximum concentration in the linear region was taken as the solubility. The solubility of **SE1** in PBS buffer was ~ 2.0 μM, while that of **1** was ~ 3.0 μM,[3] respectively.

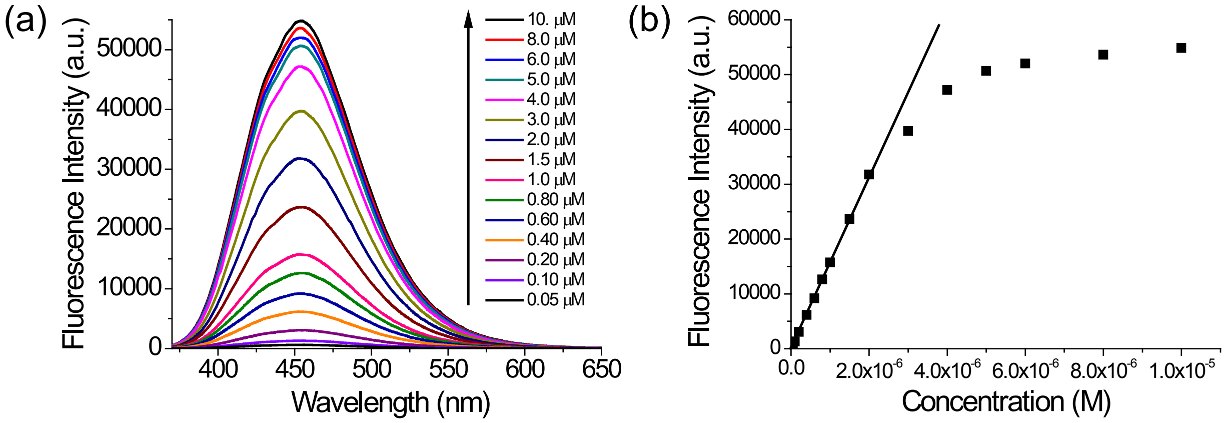

Figure S1. (a) One-photon fluorescence spectra and (b) plot of fluorescence intensity against the concentration of **SE1** in PBS buffer (10 mM, pH 7.4). The excitation wavelength was 332 nm.

Table S1. Photophysical data for **SE1** and **1** in buffer.[a]

Compound	$\lambda_{max}^{(1)}$ $(10^{-4}\,\varepsilon)$[b]	λ_{max}^{fl}[c]	Φ[d]	R_{max}/R_{min}[e]	$\lambda_{max}^{(2)}$[f]	$\Phi\delta_{max}$[g]
**SE1**	332 (2.37)	455	0.55	118	740	12
**1**	378 (2.50)	540	0.16		750	58

a) All data were measured in PBS buffer (10 mM, pH 7.4, 37 °C). b) λ_{max} of the one-photon absorption spectra in nm. The numbers in parentheses are molar extinction coefficients in $M^{-1}cm^{-1}$. c) λ_{max} of the one-photon emission spectra in nm. d) Fluorescence quantum yield, ±10%. e) Emission ratio ($F_{520\text{-}570}/F_{420\text{-}470}$) conversion factor, R_{max}/R_{min}, measured by one-photon process before and after addition of porcine liver esterase (PLE, 0.2 unit). f) λ_{max} of the two-photon emission spectra in nm. g) The peak two-photon action cross sections in GM (1 GM = 10^{-50} cm^4 s $photon^{-1}$), ±15%. Data for **1** was taken from Ref. 3.

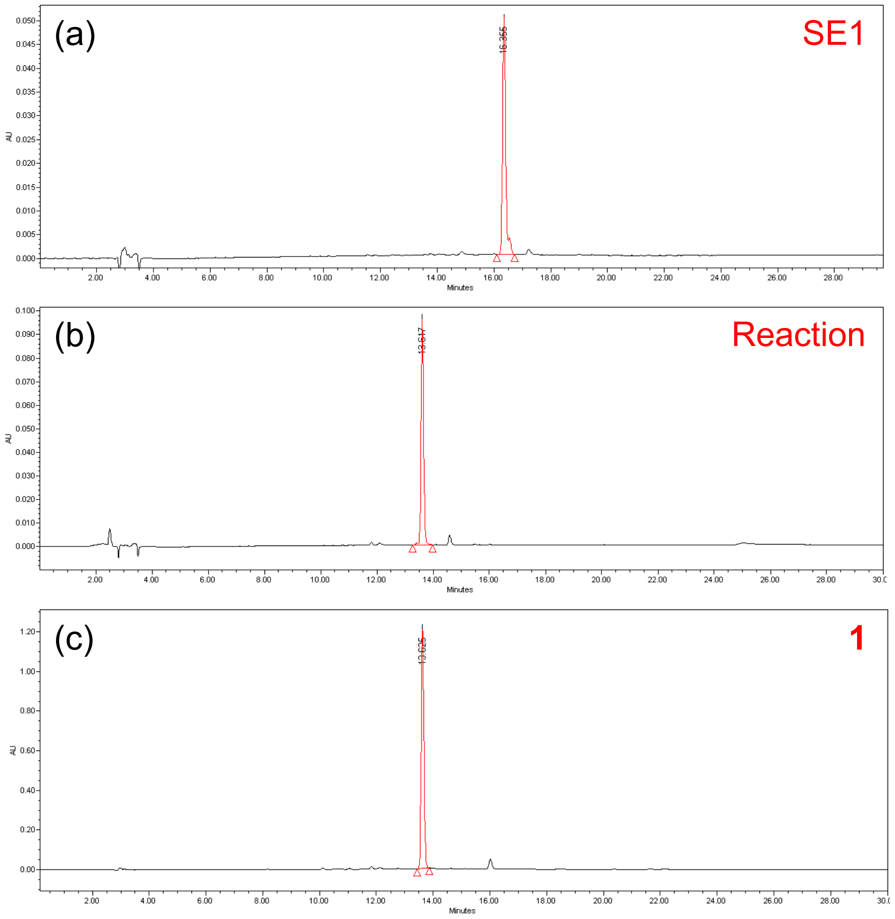

Figure S2. HPLC traces of (a) **SE1**, (b) the reaction product between **SE1** and PLE, (c) **1**. HPLC conditions: 1.0 mL/min flow rate, 5% B to 100% B over 30 min, detected at 370 nm. Solvent A is water and solvent B is acetonitrile. Peaks at 16.4 min and 13.6 min correspond to **SE1** and **1**, respectively.

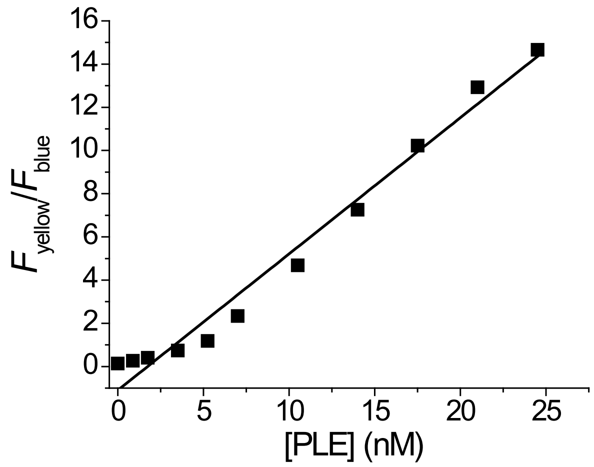

Figure S3. Plot of the F_{yellow}/F_{blue} ratios for **SE1** vs [PLE] in PBS buffer (10 mM, pH 7.4). Each data was acquired 10 min after PLE addition at 37℃. The detection limit was calculated with 3σ/k; where σ is the standard deviation of blank measurement, k is the slope in Fig. S3.[5]

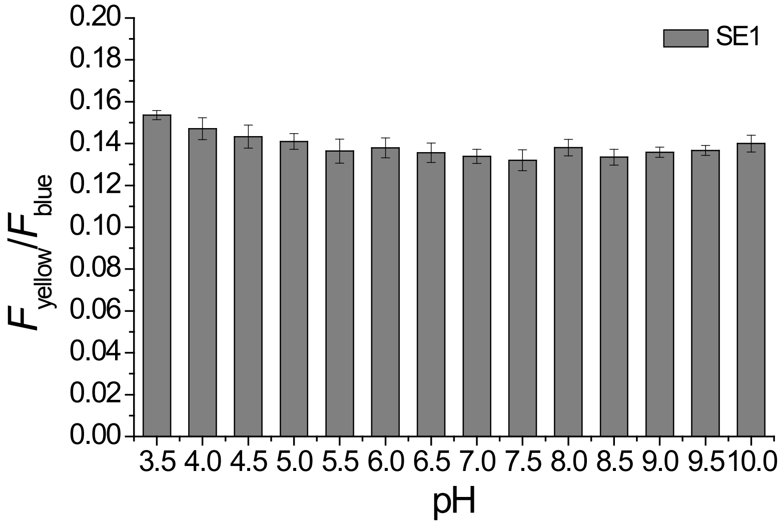

Figure S4. Effect of pH on the fluorescence intensity ratios (F_{yellow}/F_{blue}) for **SE1** in universal buffer (0.1 M citric acid, 0.1 M KH_2PO_4, 0.1 M $Na_2B_4O_7$, 0.1 M Tris, 0.1 M KCl) at 25 °C. The excitation wavelength was 373 nm (n = 3).

Table S2. Kinetic parameters for **SE1** with PLE.

Compound	K_m (μM)	k_{cat} (s^{-1})	k_{cat}/K_m (M^{-1} s^{-1})	V_{max} (nmol mg^{-1} s^{-1})
**SE1**	4.33 ± 0.22	0.890 ± 0.012	2.05(±0.140) × 10^5	4.67 ± 0.064

Stability in cell medium. Stabilities of **SE1** and FDA in cell medium were determined by monitoring the changes of fluorescence intensity with time.[6] The fluorescence intensity was collected at 540 nm (λ_{ex} = 378 nm) for **SE1**, 512 nm (λ_{ex} = 488 nm) for FDA with 5 minute intervals from 0 to 2h at 37 ºC in MEM (WelGene Inc, Seoul, Korea) medium supplemented with 10 % FBS (WelGene), penicillin (100 units/ml), and streptomycin (100 μg/mL) containing 1.0 μM **SE1** and FDA. The fluorescence intensities of the corresponding hydrolyzed compounds (1.0 μM **1** for **SE1** and 1.0 μM Fluorescein for FDA) were normalized as 1.0 in Figure S5.

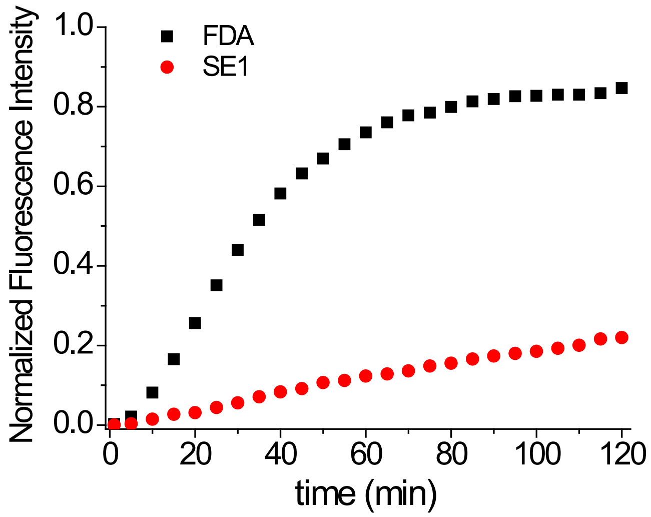

Figure S5. Comparison of the time course of the normalized fluorescence intensity between 1.0 μM **SE1** (red) and 1.0 μM FDA (black) in MEM medium containing 10% (v/v) FBS.

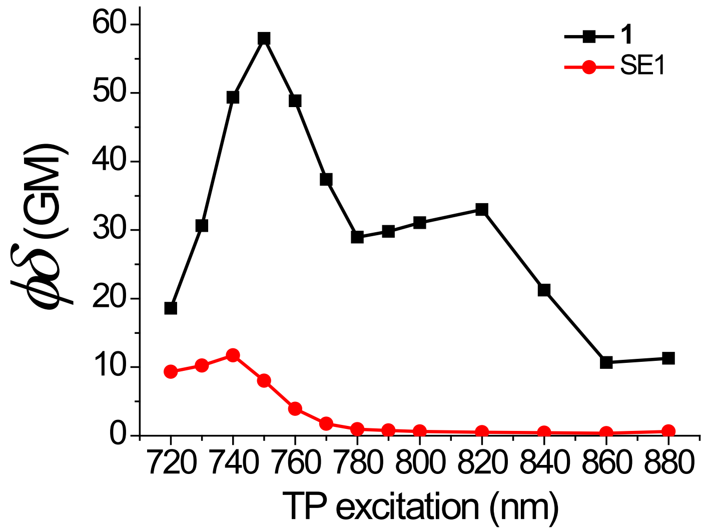

Figure S6. Two-photon action spectra of **SE1** and **1** in PBS buffer (10 mM, pH 7.4). The estimated uncertainties for the two-photon action cross section values ($\delta\Phi$) are ± 15 %. Data for **1** was taken from Ref. 3.

Photostability. Photostability of **SE1** was determined by monitoring the changes in TPEF intensity with time at three designated positions of **SE1**-labeled (2.0 μM) HepG2 cells chosen without bias (Figure S7). The TPEF intensity remained nearly the same for one hour, indicating high photostability.

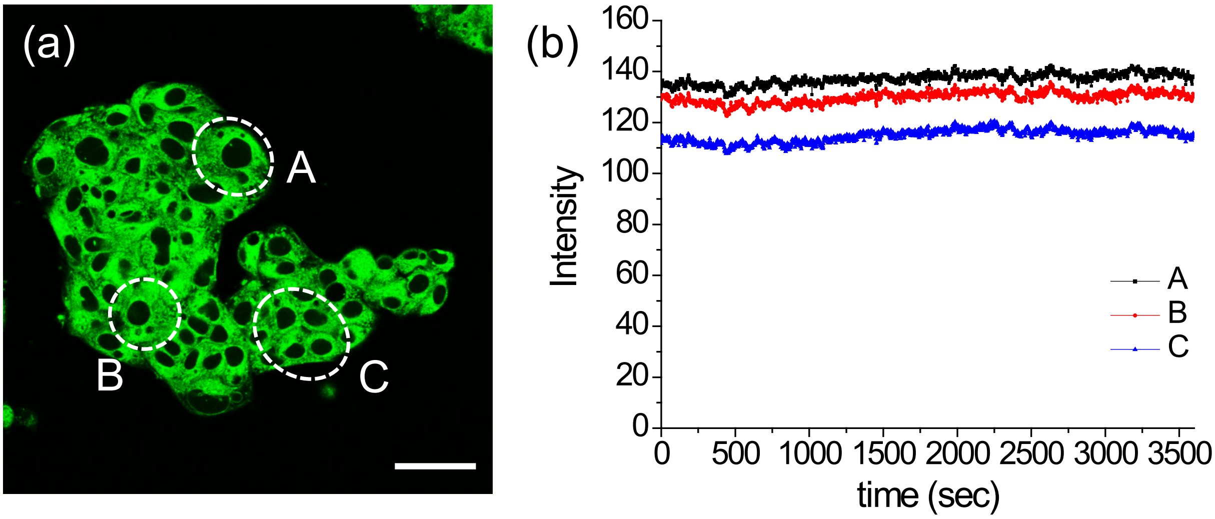

Figure S7. (a) TPM images of HepG2 cells labeled with **SE1**. (b) The relative TPEF intensity from A–C in Figure (a) as a function of time. The digitized intensity was recorded with 2.00 sec intervals for the duration of one hour using *xyt* mode. The TPEF intensities were collected at 420–570 nm upon excitation at 740 nm with femto-second pulses. Scale bar = 48 μm. Cells shown are representative images from replicate experiments (n = 3).

Cell viability. To evaluate the cytotoxic effect of **SE1** in HepG2 cells, MTS (cell Titer 96H; Promega, Madison, WI, USA) and CCK-8 kit (Cell Counting Kit-8; Dojindo, Japan) assay were performed according to the manufacture's protocol. The results are shown in Figure S8, which revealed that the **SE1** has low cytotoxicity at its different concentration in our incubation condition.

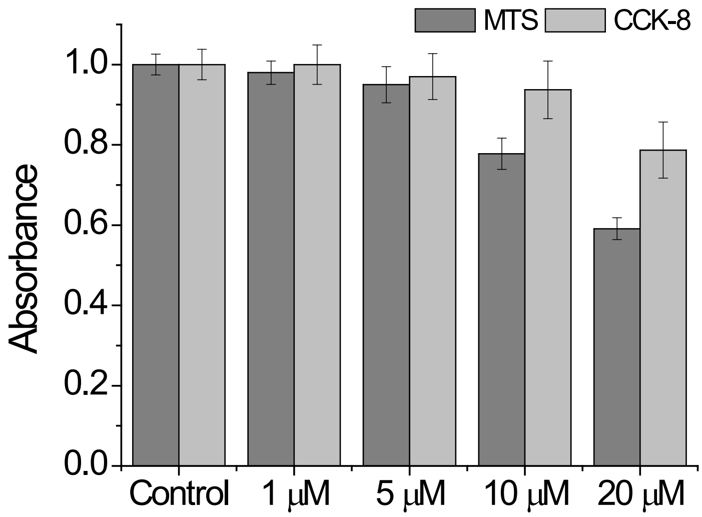

Figure S8. Viability of HepG2 cells in the presence of **SE1** as measured by using MTS and CCK-8 assays. The cells were incubated with 0–20 μM **SE1** for 2 h. Six independent experiments are performed.

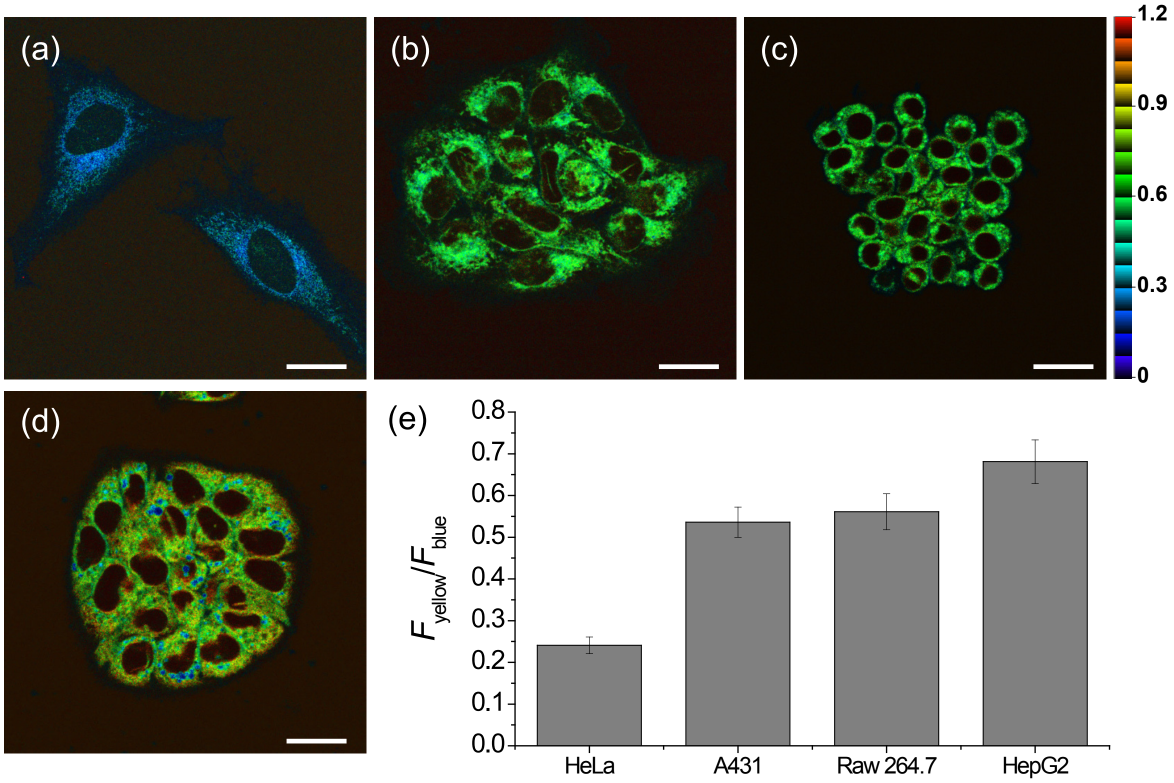

Figure S9. Pseudocolored ratiometric TPM images (F_{yellow}/F_{blue}) of (a) HeLa, (b) A431, (c) Raw 264.7 and (d) HepG2 cells incubated with **SE1** (2.0 μM) for 30 min. (e) Average F_{yellow}/F_{blue} intensity ratios in (a-d). Images were acquired using 740 nm excitation and emission windows of 420–470 nm (blue) and 520–570 nm (yellow). Scale bars = (a) 27 and (b-d) 19 μm. Cells shown are representative images from replicate experiments (n = 10).

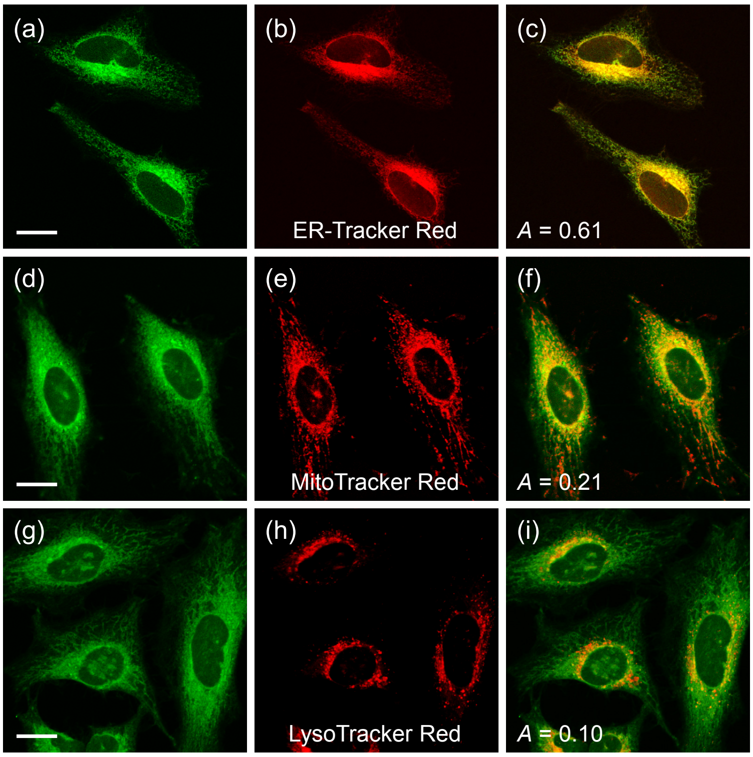

Figure S10. (a,d,g) TPM, (b,e,h) OPM images of HeLa cells co-labeled with (a,d,g) **SE1** (2 μM) and (b,e,h) organelle trackers. (c,f,i) Merged images. Excitation wavelengths for TPM and OPM are 740 nm and 552 nm, respectively. Scale bars = (a) 24 and (d, g) 20 μm.

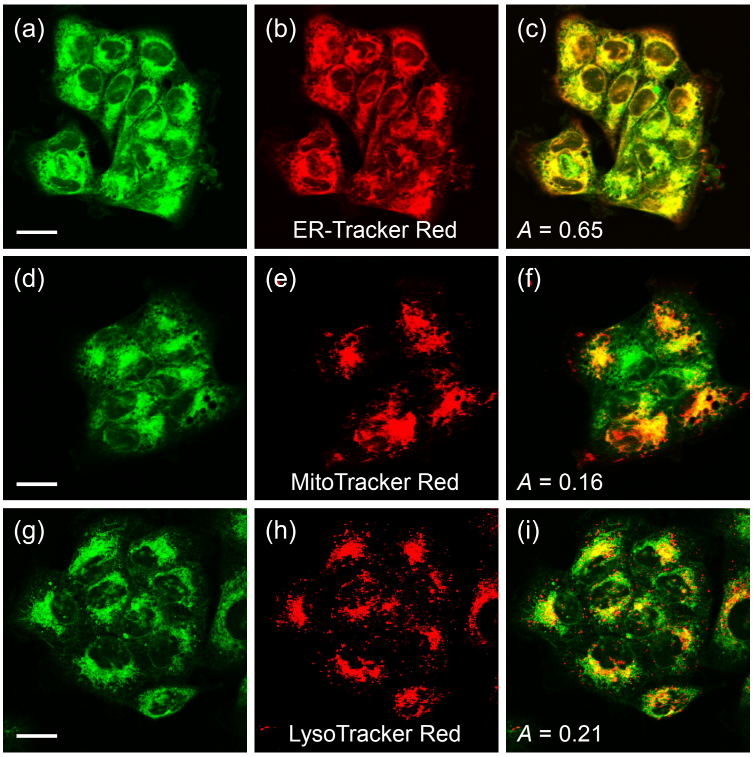

Figure S11. (a,d,g) TPM, (b,e,h) OPM images of A431 cells co-labeled with (a,d,g) **SE1** (2 μM) and (b,e,h) organelle trackers. (c,f,i) Merged images. Excitation wavelengths for TPM and OPM are 740 nm and 552 nm, respectively. Scale bars = (a, g) 21 and (d) 19 μm.

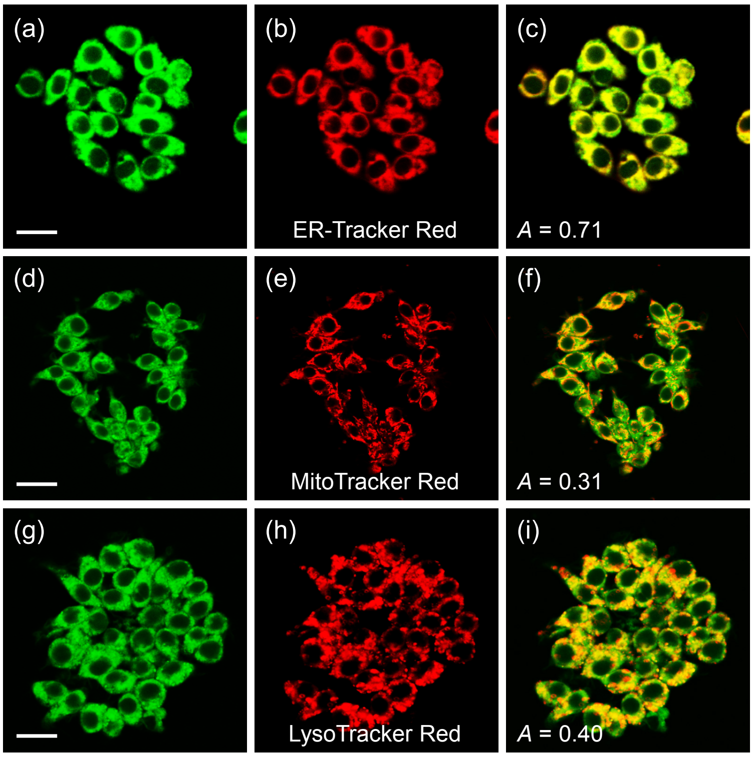

Figure S12. (a,d,g) TPM, (b,e,h) OPM images of Raw 264.7 cells co-labeled with (a,d,g) **SE1** (2 μM) and (b,e,h) organelle trackers. (c,f,i) Merged images. Excitation wavelengths for TPM and OPM are 740 nm and 552 nm, respectively. Scale bars = (a, g) 15 and (d) 24 μm.

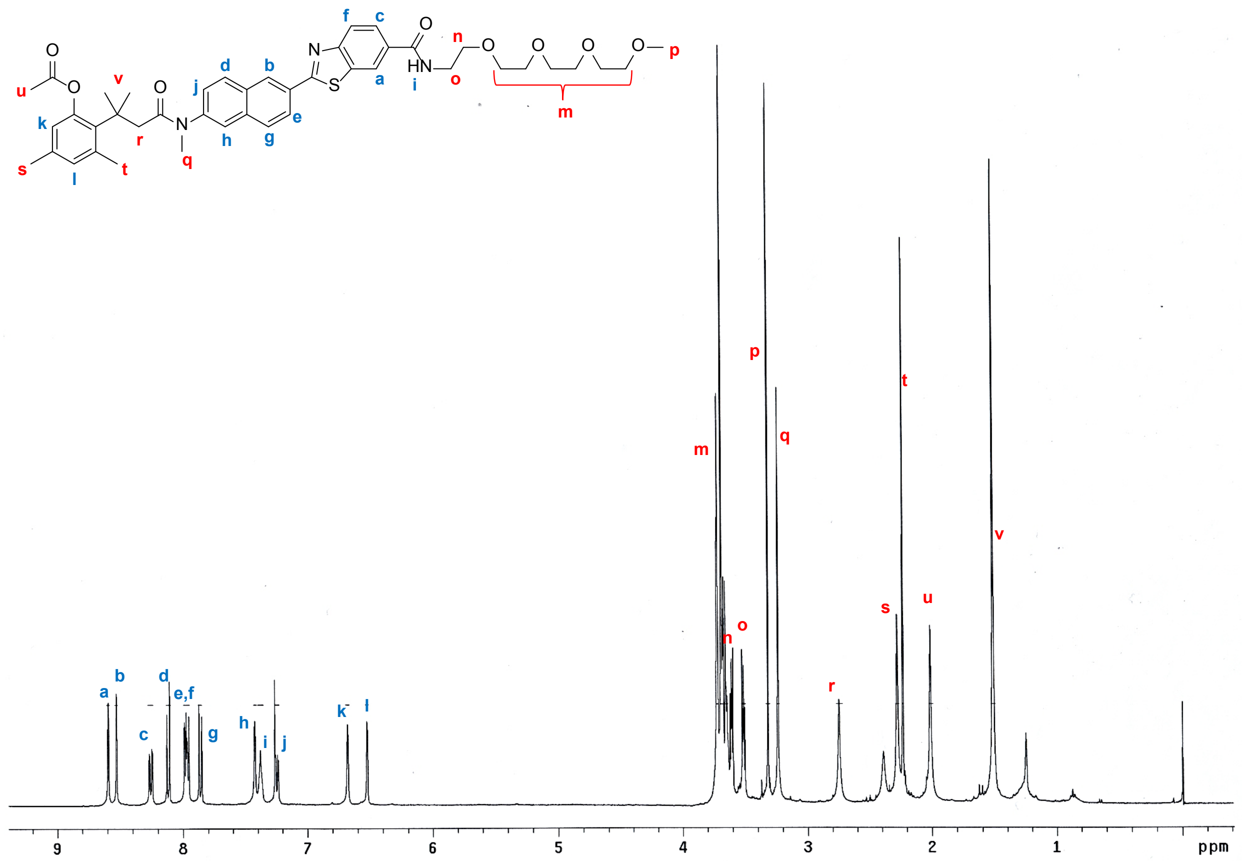

Figure S13. ^{1}H-NMR spectrum (400 MHz) of **SE1** in $CDCl_3$

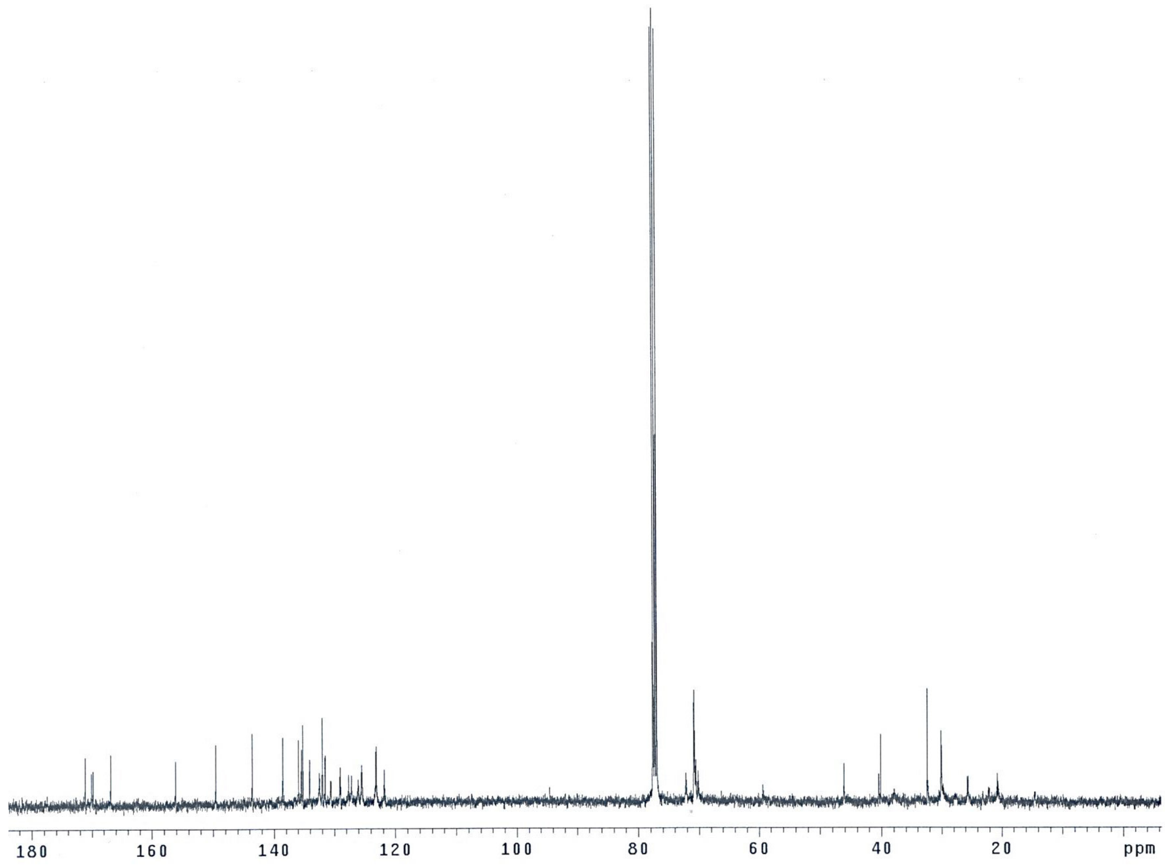

Figure S14. ^{13}C-NMR spectrum (100 MHz) of **SE1** in $CDCl_3$

1) SE1 *with NBA (POS)*

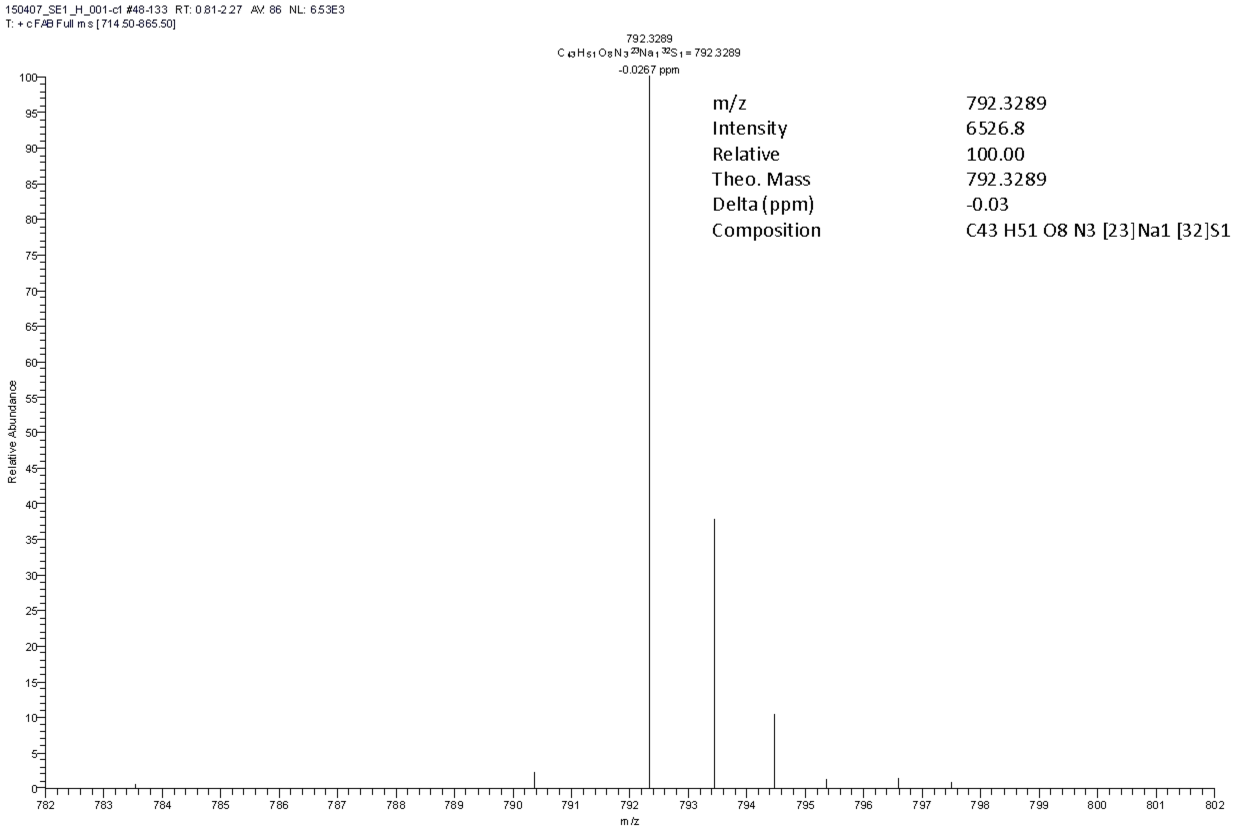

Figure S15. HRMS spectrum of **SE1**

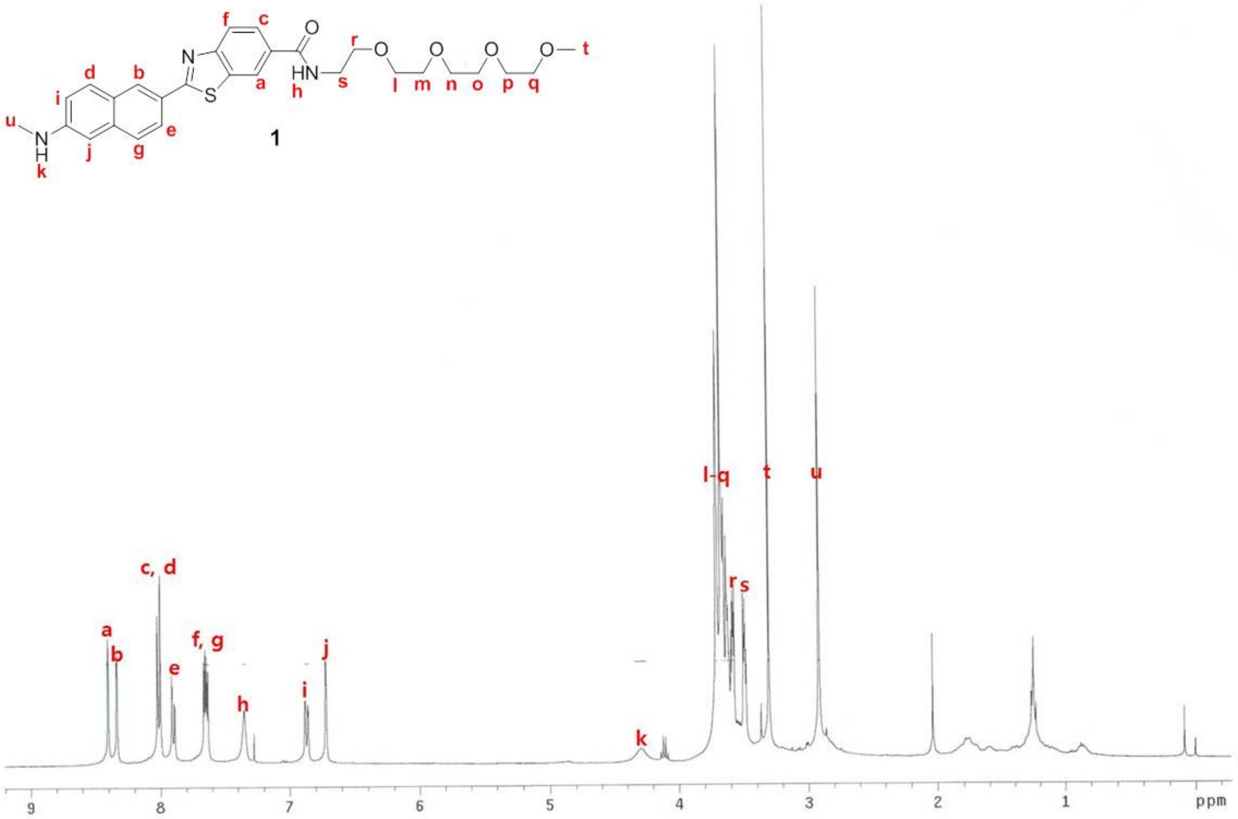

Figure S16. ^{1}H-NMR spectrum (400 MHz) of **1** in $CDCl_3$

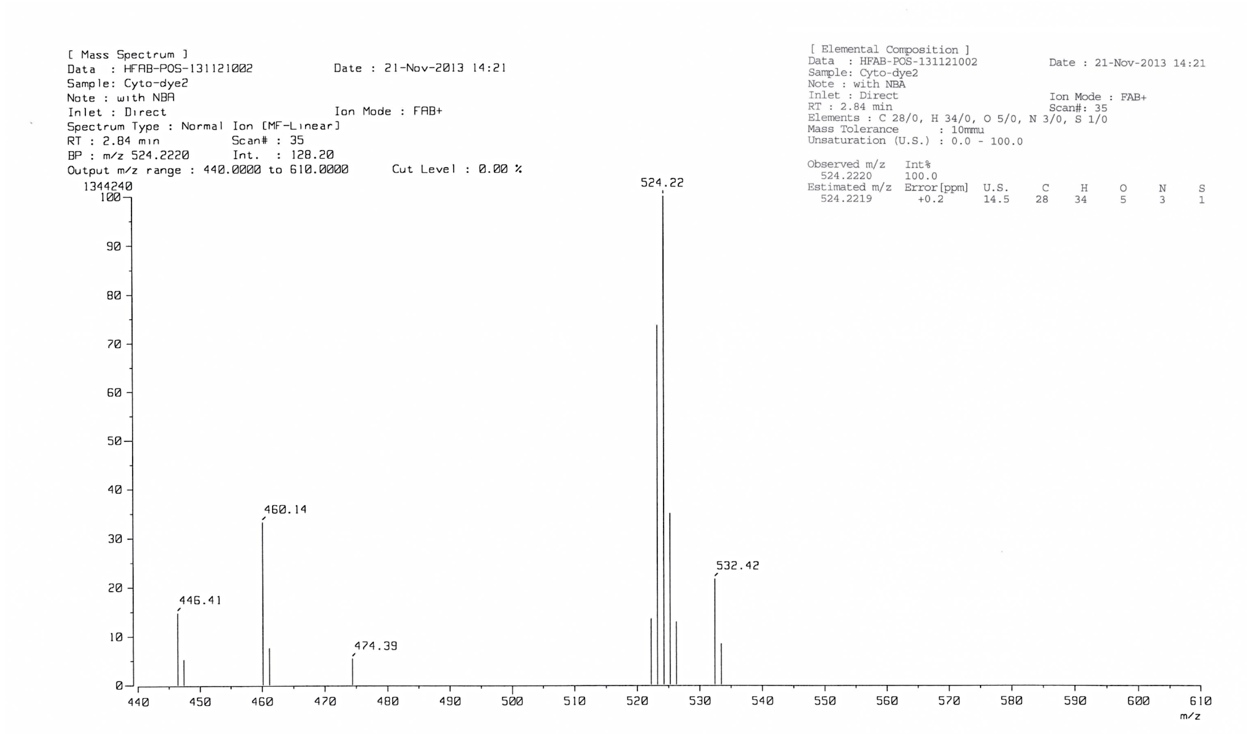

Figure S17. HRMS spectrum of **1**

References

1. C. S. Lim, G. Masanta, H. J. Kim, J. H. Han, H. M. Kim, B. R. Cho, *J. Am. Chem. Soc.,* 2011, **133**, 11132.
2. A. Tuchscherer, D. Schaarschmidt, S. Schulze, M. Hietschold, H. Lang, *Eur. J. Inorg. Chem.,* **2011**, 4421.
3. H. W. Lee, C. H. Heo, D. Sen, H. O. Byun, I. H. Kwak, G. Yoon, H. M. Kim, *Anal. Chem.,* 2014, **86**, 10001.
4. H. M. Kim, H. J. Choo, S. Y. Jung, Y. G. Ko, W. H. Park, S. J. Jeon, C. H. Kim, T. Joo, B. R. Cho, *Chembiochem,* 2007, **8**, 553.
5. V. Thomsen, D. Schatzlein, D. Mercuro, *Spectroscopy,* 2003*,* **18***,* 112.
6. L. D. Lavis, T. Y. Chao, R. T. Raines, *Chem. Sci.,* 2011, **2**, 521.
